# A systematic review and functional bioinformatics analysis of genes associated with Crohn’s disease identify more than 120 related genes

**DOI:** 10.1186/s12864-022-08491-y

**Published:** 2022-04-13

**Authors:** Debora Garza-Hernandez, Maricruz Sepulveda-Villegas, Jose Garcia-Pelaez, Raul Aguirre-Gamboa, Peter L. Lakatos, Karol Estrada, Manuel Martinez-Vazquez, Victor Trevino

**Affiliations:** 1 Tecnologico de Monterrey, Escuela de Medicina, Cátedra de Bioinformática, Av. Morones Prieto No. 3000, Colonia Los Doctores, 64710 Monterrey, Nuevo León Mexico; 2 Instituto de Investigação e Inovação em Saude-i3S, Universidade do Porto, Porto, Portugal; 3 Ipatimup, Institute of Molecular Pathology and Immunology at the University of Porto, Porto, Portugal; 4 Department of Human Genetics, University of Chicago, Chicago, USA; 5 McGill University Health Centre, Division of Gastroenterology, IBD Centre, Montreal General Hospital, 1650 Ave. Cedar, D16.173.1, Montreal, QC H3G 1A4 Canada; 6 Graduate Professional Studies, Brandeis University, Waltham, MA 02453 USA; 7 Tecnologico de Monterrey, Instituto de Medicina Interna, Centro Médico Zambrano Hellion, Av. Batallón de San Patricio No. 112, Colonia Real San Agustín, 66278 San Pedro Garza García, Nuevo León Mexico; 8 Tecnologico de Monterrey, The Institute for Obesity Research, Integrative Biology Unit, Eugenio Garza Sada 2501 Avenue, 64849 Monterrey, Nuevo Leon Mexico

**Keywords:** Crohn’s disease, Inflammatory bowel disease, Genes, Functional variants, Mutations

## Abstract

**Background:**

Crohn’s disease is one of the two categories of inflammatory bowel diseases that affect the gastrointestinal tract. The heritability estimate has been reported to be 0.75. Several genes linked to Crohn’s disease risk have been identified using a plethora of strategies such as linkage-based studies, candidate gene association studies, and lately through genome-wide association studies (GWAS). Nevertheless, to our knowledge, a compendium of all the genes that have been associated with CD is lacking.

**Methods:**

We conducted functional analyses of a gene set generated from a systematic review where genes potentially related to CD found in the literature were analyzed and classified depending on the genetic evidence reported and putative biological function. For this, we retrieved and analyzed 2496 abstracts comprising 1067 human genes plus 22 publications regarding 133 genes from GWAS Catalog. Then, each gene was curated and categorized according to the type of evidence associated with Crohn’s disease.

**Results:**

We identified 126 genes associated with Crohn’s disease risk by specific experiments. Additionally, 71 genes were recognized associated through GWAS alone, 18 to treatment response, 41 to disease complications, and 81 to related diseases. Bioinformatic analysis of the 126 genes supports their importance in Crohn’s disease and highlights genes associated with specific aspects such as symptoms, drugs, and comorbidities. Importantly, most genes were not included in commercial genetic panels suggesting that Crohn’s disease is genetically underdiagnosed.

**Conclusions:**

We identified a total of 126 genes from PubMed and 71 from GWAS that showed evidence of association to diagnosis, 18 to treatment response, and 41 to disease complications in Crohn’s disease. This prioritized gene catalog can be explored at http://victortrevino.bioinformatics.mx/CrohnDisease.

**Supplementary Information:**

The online version contains supplementary material available at 10.1186/s12864-022-08491-y.

## Background

Inflammatory bowel diseases (IBD) comprise Crohn’s disease (CD) and ulcerative colitis (UC), which are inflammatory diseases of the gastrointestinal tract with an unknown etiology [1]. Common symptoms of CD include abdominal pain, fever, diarrhea, and bleeding, depending on disease severity [2]. Disease complications can lead to bowel disability and sometimes to surgery [3]. CD is more frequent among industrialized nations such as North America, with a reported incidence of 6.3 to 23.8 per 100,000, and Western Europe, with 1.9 to 10.5 per 100,000 people [4, 5].

Therefore, in addition to common risk factors for CD, the contribution of genetic factors in CD has been considered highly relevant. This contribution is based on the fact that family history can influence the presence of the disease, with a higher risk for siblings with a relative risk of 13 to 36 times [6]. In fact, heritability estimates for Crohn’s disease from pooled twin studies have been reported to be 0.75 [7].

As with many other complex traits [8], several CD related-genes have been identified through the use of linkage-based studies, candidate gene association studies (i.e., transmission disequilibrium tests), and high coverage technologies such as DNA arrays and next-generation sequencing (NGS) [9, 10]. Among well-known risk genes for CD are *NOD2*, *IL23R*, and *ATG16L1* [11], which are involved in inflammation and the immune system’s response [11, 12].

In addition to candidate genes association studies, the implementation of high coverage technologies, such as NGS, has improved the molecular diagnostic yield of complex diseases such as CD. These strategies typically make use of phenotype-specific panels containing genes that are known to confer susceptibility for a complex disease [13, 14]. Specifically, for CD there have been attempts to test for genetic susceptibility for treatment response and prognosis in CD patients [15, 16]. Genome-wide association studies (GWAS) have also made large contributions identifying more than 130 genes [17–19]. From these, genome-wide polygenic risk scores (PRS) aim to identify individuals at significantly increased risk. For CD, PRS from over 200 loci, yields an estimate of 8% of variance explained and an AUC around 0.7 [20].

A comprehensive collection of genes for CD is lacking, which complicates further functional analyses and overall understanding. Different aspects of CD have been reviewed, including inflammatory drugs and risk of exacerbation [21], pouch incidence [22], prognostic factors [23, 24], and biomarkers for surgery outcomes [25]. Nevertheless, to our knowledge, there is a lack of functional analyses and systematic reviews analyzing all known genes or variants associated with CD susceptibility. We conducted this compilation by first classifying each gene based on the genetic evidence reported and then functionally analyzing those genes. We hope this collection of genes and functional analysis might help for further understanding of the disease etiology.

Starting from a Pubmed query, we systematically curated 2496 abstracts following recommended methodologies to identify and functionally classify genes associated with CD. To further support our findings, we provided functional analyses of the identified genes. We show that although most of the research in CD revolves around a group of well-known genes, our systematic curation review identified 126 genes with a sufficient level of associative evidence.

## Methods

Based on our previous work [26], we collected abstracts related to genetic variations in CD from the PubMed repository. The following review process adheres to the Preferred Reporting Items for Systematic Review and Meta-Analysis (PRISMA) 2020 guidelines [27]. The abstracts were manually revised, curated, and annotated for each identified gene by using the PubTerm web tool [28]. Each gene was designated to a specific category based on the reported genetic alteration or evidence related to CD. The details are described in the next sections.

### Abstract collection

Only original research papers published in English were considered. The search strategy comprised three basic terms: (1) Crohn’s disease, (2) genetic variations, and (3) focus on humans. Thus, the following query was used: crohn*[TIAB] AND (mutation*[TIAB] OR polymorphism*[TIAB] OR variant*[TIAB]) NOT review[Publication Type] NOT mouse[TIAB] NOT mice[TIAB]. The query was performed during 2019 and updated in January 2020. We used PubTerm to curate and annotate abstracts per gene, previously used in pulmonary arterial hypertension and vitamin D levels [26, 29]. Additionally, we reviewed GWAS publications as described below.

### Definition of gene categories

We defined categories to annotate the genes identified based on the genetic evidence related to CD ordered by importance as: (i) *Experimental evidence of a variant,* when experimental evidence of specific sequence variants is shown for CD; (ii) *GWAS evidence within gene,* if sequence variants or single nucleotide polymorphisms (SNP) were found within a gene region in GWAS; (iii) *Genetic evidence in treatment response,* when experimental evidence of sequence variants were associated with response to CD treatments; (iv) *Genetic evidence in related complications,* if experimental evidence of sequence variants were associated with CD complications; (v) *Other genetic alterations,* when no specific sequence variant information was provided (e.g. haplotypes, SNP at intergenic regions, uncertain locus); (vi) *Genetic evidence in a related disease,* when experimental evidence of sequence variants is shown for other related diseases rather than specifically to CD; (vii) *Related but not variant reported*, if no genetic evidence is shown but there is a biological relationship mentioned between the gene and the disease (e.g. gene expression changes); (viii) *Negative evidence*, if the gene is properly annotated but the conclusion of the research was a not causal relationship; (ix) *Unrelated*, if the gene is correctly annotated but there is no mention of the causal association of the gene with the disease; (x) *Annotation error*, if the gene is not related to CD due to nomenclature errors, inaccurate disease, and other diverse errors. *SNX20* gene was added manually, as our search identified a paper with evidence of its association with CD, but the *SNX20* gene symbol was not correctly identified by the tools used.

### Curation and categorization per gene

After the abstracts were retrieved from PubMed into the PubTerm tool [28], we filtered for only human genes, and each gene was subsequently reviewed. All the abstracts organized per gene were carefully read and analyzed until enough evidence was convincing to assign the gene to a specific category, or all abstracts were carefully read. If two categories apply, the category with more relevant genetic information was used. The full text was reviewed when necessary, commonly when a sequence variant was not clear, uncertain, or in negative cases. The critical sentence in the abstract and the PubMed ID was added to the PubTerm notes in every gene analyzed to support the decision made, which is available electronically within PubTerm, as shown below. Most genes were reviewed by two authors. All results can be obtained from Supplementary Table 1 and PubTerm (http://victortrevino.bioinformatics.mx/pubterm) using the user “vtrevino@tec.mx” and project “Crohn_s Disease”. In addition, to facilitate rapid revision, we provide a summary list at http://victortrevino.bioinformatics.mx/CrohnDisease .

### GWAS variants revision

A search for GWAS studies was performed at GWAS Catalog [30] in order to retrieve variants that were not mentioned and indexed directly into PubMed abstracts and full texts. For this, only publications with reported associations specifically for CD were used. This was done using the search term Crohn’s disease trait with EFO_0000384. Also, a comparative search was performed at the Open Targets platform [31], which integrates public domain data to enable finer target identification and prioritization for a given disease. For comparative purposes, only the genetic associations data type was used. This data comes from a Linkage-disequilibrium expansion and fine mapping of GWAS curated associations. Thus, it aims to identify the most likely causal variant linked to the GWAS detected variant. If a gene found in GWAS and PubMed, the higher categorical evidence was kept.

### Identification and annotation of variants

To further support our findings, the variants were also reviewed in ClinVar [32]. For genes not reported in ClinVar, a manual annotation approach was performed by using the information from the original publication. The list of variants and their respective transcript or “rs ID numbers” (for SNPs) is presented as supplementary information.

### In-silico functional analysis of genes

For the CD confirmed genes (*n* = 126), a functional analysis was carried out using DAVID [33]. This tool performs an over-representation test to determine, from an input set of genes, if the number of genes appearing in a biological pathway or biological term is not random. In such a case, the gene set is said to be tightly associated with the term. We used a hierarchical clustering approach to group the biological terms obtained from DAVID for comparison and summarization purposes. The functional analysis performed consisted of Gene Ontology terms (including *cellular component*, *biological processes*, and *molecular functions* [34, 35], KEGG pathways, and related diseases from the genetic association database (GAD) [36]. The criteria for clustering terms consisted of selecting those terms statistically significant (after Bonferroni correction for *p* < 0.05) and that involved a large number of genes (≥12 for diseases, ≥ 10 for GO and KEGG). Manual merging was also performed to group similar concepts and hence facilitated the interpretation. Because many highly related terms were observed, the significant terms were grouped by similarity using hierarchical clustering separately for GO and KEGG and by groups of similar disorders or diseases. Groups were generated by averaging the presence of the gene among the diseases/terms merged in the group.

Additionally, we used the Gene Network v2.0 tool [37] to identify Human Phenotype Ontology (HPO) clusters [38]. We selected the most distinguishable clusters based on co-regulation scores across public RNA-Seq samples and ran a phenotype analysis for each selection. Only terms with a significant enrichment (Bonferroni *p* < 0.05) were selected.

For the differential expression, the 126 genes associated with CD were analyzed within the recently published Gene Expression Omnibus dataset [39] GSE111889, which shows recent data from UC and CD compared to normal ileum and colon. Differential expression was performed separately for CD, UC, and tissue. A linear model regression with sex correction was fitted for each analysis. Differentially expressed genes (DE) were selected after a false discovery rate correction < 0.05 [40].

Gene-drug interactions were analyzed using the drug-gene interaction database (DGIdb) [41] using the default parameters.

### Benchmark on genes present on commercial panels and identified by GWAS

To show the relevance of the extracted data and possible applications, a comparison was performed between the genes extracted from our curation process and the Genetic Test Registry (GTR) [42]. For GTR, diagnosis panels for CD and related diseases were tested. The clinical panels used for comparison were searched for the IBD1: Crohn Disease, the criteria for selecting the tests consisted of selecting only specific diagnostic tests for CD or inflammatory-related diseases. A general web search was also performed with a search strategy comprising the queries “*Crohn’s disease testing panels*”, “*Crohn’s disease genetic diagnosis panels*”, and “*Crohn’s disease diagnosis commercial panels*”.

## Results

### Classification and identification of CD genes

**Fig. 1:**
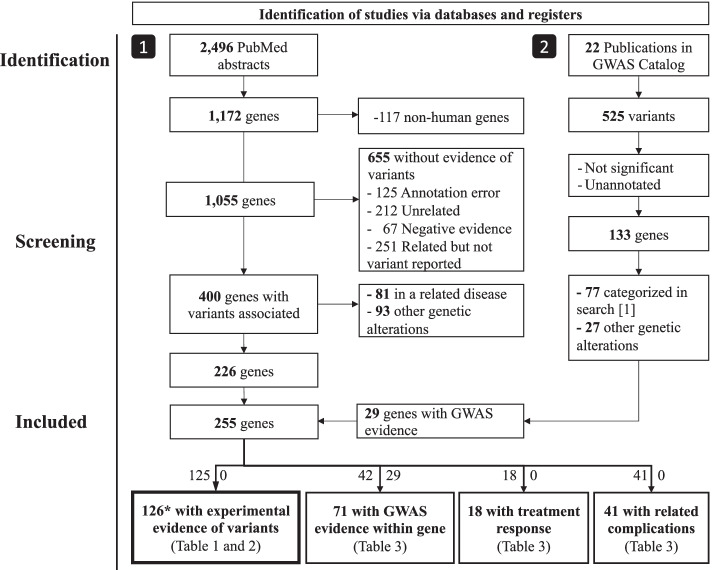
Summary of categorized genes for Crohn’s disease. The numbers at left in arrows at the bottom represent the genes from search [1], while the numbers at right correspond to search [2]. *SNX20 was added manually

The PRISMA flow chart for the selection of studies and genes is shown in Fig. 1. The PRISMA checklist is provided as supplementary information (File S1). The PubMed search imported into PubTerm identified 2496 articles, which referred to 1172 genes. The genes were reduced to 1055 after filtering for human genes (Fig. 1, Table S1). Then, each gene was carefully curated, annotated, and categorized as described in methods. The curation revealed that 400 genes were somehow potentially related to CD while 655 genes were not related due to annotations errors, the gene mention was casual, no association was found, or no evidence of variation was shown (mainly in subsequent gene expression changes). From the 400 genes, 81 were finally categorized as associated with a *related disease* such as IBD in general, UC, familial diarrhea syndrome, colorectal cancer, or chronic lymphocytic leukemia. Ninety-three genes were classified to *other genetic alterations* because its gene was uncertain, which included genes identified through haplotypes or intergenic regions in GWAS. Thus 226 genes were confirmed as associated with CD from this curation (Fig. 1).

We also used GWAS Catalog [30] as a source of gene information. From 22 publications for CD risk, we obtained the 525 comprising variants. Variants were further filtered by removing those whose tagged gene was not reported, were not significant, or the gene or variant was duplicated, leaving only 133 genes (Table S2). From these, 77 were already categorized in the PubMed curation described above. Thus 56 genes were added to our list of genes, 27 intergenic variants were assigned to *other genetic alterations,* and 29 to *GWAS evidence within gene*. A list of the considered PubMed abstracts is shown in Supplementary Information (files S2 and S3). In summary, we identified 256 genes associated with diverse aspects of CD (Fig. 1).

A total of 126 genes were found to have *experimental evidence of variants* in CD. The top 26 genes of this category mentioned in more than 15 abstracts are shown in Table 1, while the genes with less than 15 abstracts are summarized in Table 2 and detailed in the Supplementary Information (Table S4). The topmost frequent genes for this category are well-known for their association with CD [11, 12], such as *NOD2*, *TNF*, *IL23R*, *ATG16L1, TLR4, IL10, SLC22A4, SLC22A5*, and *IRGM* (Table 1).

**Table 1:** The subset of top genes with experimental variants associated with CD (abstracts > 15)

***Gene***	***Abstracts***	***Panels***	***GWAS catalog studies***	***ClinVar***
*NOD2*	751	16	8	Yes
*TNF*	156	0	0	Yes
*IL23R*	148	1	6	Yes
*ATG16L1*	128	1	5	Yes
*TLR4*	88	0	0	No
*IL10*	86	2	1	Yes
*SLC22A4*	73	0	0	Yes
*IRGM*	60	1	3	Yes
*SLC22A5*	50	0	0	Yes
*TNFSF15*	45	0	4	No
*NOD1*	41	0	0	No
*IL6*	41	4	0	Yes
*IL1B*	40	0	0	No
*STAT3*	37	1	2	No
*NFKB1*	36	0	0	No
*DLG5*	35	0	0	No
*IL12B*	34	0	0	No
*ABCB1*	33	1	0	Yes
*KRAS*	33	0	0	No
*PTPN22*	31	0	0	No
*IL1RN*	29	0	0	No
*IL23A*	28	0	0	No
*CD14*	28	0	0	No
*PTPN2*	24	0	0	No
*IL10RA*	20	2	0	Yes
*NLRP3*	19	0	0	No
*MEFV*	20	1	0	No
*IL4*	18	0	0	No
*NKX2–3*	17	0	0	No
*ICAM1*	16	0	1	No
*IFNG*	16	0	0	No
*TLR9*	16	0	0	No

**Table 2:** Genes with experimental variants associated with CD mentioned by less than 15 abstracts. Details are provided in Table S1. * denotes manual addition

*ACE*	*CFTR*	*EPX*	*IL10RB*	*MST1*	*PTGS2*	*TLR6*
*AGER*	*CLEC2D*	*ERAP2*	*IL16*	*MTRR*	*REEP6*	*TNFAIP3*
*AGT*	*CREM*	*FAS*	*IL18*	*MUC2*	*SFTPD*	*TNFRSF1A*
*APOE*	*CSF1R*	*FUT2*	*IL27*	*MUC3A*	*SLC11A1*	*TNFRSF1B*
*ATG16L2*	*CSF2RB*	*GC*	*IL4R*	*MYO9B*	*SLC15A1*	*TNFSF8*
*BDKRB1*	*CX3CR1*	*GSTT1*	*IRF1*	*NAT2*	*SLC22A1*	*TRAIP*
*BPI*	*CXCL16*	*HLA-DQA2*	*IRF5*	*NCF2*	*SLC39A8*	*UCP2*
*BTNL2*	*CXCR4*	*HLA-G*	*JAK2*	*NCF4*	*SLCO3A1*	*ULK1*
*CALCOCO2*	*CYP2A6*	*HNF4A*	*KCNN4*	*NFKBIA*	*SMAD3*	*XIAP*
*CARD9*	*DEFB1*	*HNRNPD*	*KRT8*	*NOS2*	SNX20*	*ZNF365*
*CCR2*	*DLG1*	*HSPA1L*	*MAGI3*	*ORMDL3*	*TCN2*	
*CCR5*	*DMBT1*	*HSPA4*	*MAP3K8*	*POU5F1*	*TIMP1*	
*CD24*	*DNAH12*	*IFNA10*	*MIF*	*PPARG*	*TLR1*	
*CD40LG*	*DUOX2*	*IFNA4*	*MLN*	*PTEN*	*TLR5*	

Besides the above 126 genes (Tables 1 and 2), we also found 71 genes associated with CD that were categorized as *GWAS evidence within gene* where an SNP is located within genomic coordinates, either an intron or exon (Table 3). Additionally, 18 genes were found to be specifically associated with *treatment response* in CD and 41 related to *disease complications* (Table 3).

**Table 3:** Other genes associated with CD for diverse categories. * Retrieved from GWASCatalog. + Retrieved from a panel

***Category***	***Feature***	***Genes*** (Abstracts)
***GWAS evidence within the gene***	SNP within gene associated to CD from GWAS	LRRK2 (19), STAT4 (14), IFNGR2 (3)*, CYLD (2), CLEC16A (2), LY75 (2), TLR8 (2), ZMIZ1 (2), CXCL12 (2), ZAP70 (2)+, TLR10 (2), PER3 (2), MTMR3 (2), MAGI1 (2), CD40 (2)*, TAB1 (1), CDYL2 (1), ELF1 (1)*, NLRP11 (1), IL2RB (1), MAP3K1 (1), TIMMDC1 (1), PDE2A (1), PRKCQ (1), SLC22A23 (1), TLE1 (1), TRPM2 (1), MORC4 (1), CYP4F2 (1), CLCA2 (1), SLC23A1 (1), GCKR (2)*, IL18RAP (4)*, BRD2 (2)*, INAVA (1)*, IL2RA (4)*+, SP140 (1)*, ITLN1 (1)*, BACH2 (4)*, GPR65 (2)*, IL1RL1 (4)*, PUS10 (1)*, ANKRD55*, OSMR*, CDH13*, DENND1B*, DNMT3A*, FOSL2*, JAZF1*, KSR1*, LPP*, TAB2*, NDFIP1*, NFATC1*, PLCL1*, RFT1*, RSPO3*, SLAMF8*, THADA*, UBE3D*, ADCY3*BANK1*,BSN*,CDC37*, DAP*,FCGR2A*,HORMAD2*,PLA2G4A*,SMURF1*,TRAF3IP2*,TSPAN14*
***Genetic evidence in treatment response***	Azathioprine	*TPMT* (41), *ITPA* (4), GSTA1 (1)
	Anti-TNF	*TLR2* (26), *IFNGR1* (2), *TBX21* (2)
	Infliximab	*FCGR3A* (11), *IL17F* ^+^ (7), *CASP9* (7), *ADAM17* ^+^ (3), *TRAF3IP2* (2), *FCGR1A* (1), *TNFAIP6* (1), ZNF133 (1)
	Corticosteroid dependency and resistance	*NR3C1* (5)
	Thiopurine	*NUDT15* (7), *NAT1* (2)
	Immunosuppressive therapy necessity	*IL1R1* (4)
***Genetic evidence in related complications***	Surgical intervention	*HSPA2* (6), *CHRNA5* (1), *CNTF* (1), *MMP9* (1), *TSPAN14* (1), *SMURF1* (1)
	Structuring behavior /Aggressive disease progression	*SERPINE1* (4), *IDO1 (2)*, *SELL* (2), *HLA*-*DOA* (1), *CSF2RA* (1), *CYBA* (1), *FAAH* (1), *ZBTB44* (1), *CACNA1E* (1), *XPO1* (1), *KIAA1614* (1), *SULF2* (1)
	Food intolerance	*FOXO3* (3),
	(mustard, ginger, tomatoes, wasabi)	
	TNF production	*NLRP12* (3)
	Granuloma formation	*ATG4A* (2), *ATG2A* (1), *ATG4D* (1), *FNBP1L* (1)
	Develop CD before 40 years of age	*CNR1* (2)
	Bone mineral density	*COL1A1* (2)
	Ileal CD	*FUT3* (2), *MIR196A2* (2), *MIR122* (1)
	Variation of GMSI level	*RCL1* (1)
	Pouch outcome	*DAGLB* (1)
	Vitamin D levels	*SCUBE3* (1), *PHF11* (1)
	Fibrostenotic CD	*PRPF31* (1)
	Favorable disease recurrence	*TIMP2* (1), *CYP26B1* (1)
	Tuberculosis and CD	*IL22RA1* (1)
	Stenotic complications	*MMP3* (1), *MMP1* (1)
	Linear growth affected	*DYM* (1)
	Colon location in CD	*MIR124*–*1* (1)

### Location of the functional variants

Of the 126 genes corresponding to the categories of *experimental evidence of variants* plus 71 genes with *GWAS evidence within gene,* only 17 genes (< 12%) were found to be annotated in ClinVar [32]. In this context, to support our systematic categorization, a supplementary file is provided with the information referring to the location of the variants that were not found in ClinVar (Tables S4 and S5). This information was revised and obtained either by the original paper or by the information related to the SNP reported at dbSNP [43].

### In-silico functional analysis

To provide an overview of the 126 genes with experimental evidence to CD, a functional bioinformatics analysis was performed [33, 44]. For this, we assessed whether the genes prioritized in our study are indeed statistical and biologically relevant. We performed gene set enrichment analysis in multiple databases containing different biological terms, including pathways (KEGG), diseases (GAD), and gene ontology (GO) terms. Detailed results of all enriched gene sets are present in the Supplementary Information. Because the number of significant terms was high, repetitive, and difficult to interpret, we grouped the terms by biological and genetic similarity (see Methods).

**Fig. 2:**
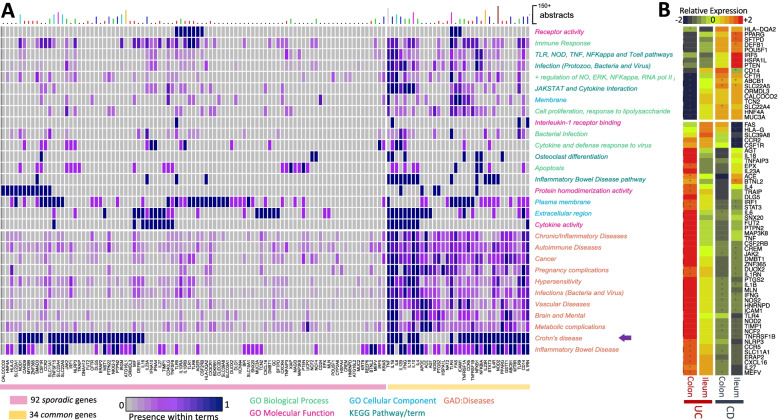
Functional and expression analysis of CD-associated Genes. **A**The y-axis comprises the GO, KEGG, and disease terms. The x-axis comprises the 126 genes used for the analysis. The heat map show values from 0 to 1, corresponding to the average presence of a gene within all terms merged in each group. *Diseases* include 10 groups, autoimmune (*Lupus Erythematosus Systemic* (LES), *Psoriasis (PS)*, *Diabetes type 1* (DT1), *Vitiligo* and *Arthritis Rheumatoid* (RA), Chronic/Inflammatory diseases (*Psoriasis, Endometriosis, Sarcoidosis, and Cystic fibrosis*), infections (*Leprosy, Tuberculosis, Sepsis, Dengue, Hepatitis, and HIV*), cancer (*Meningioma, cervical, lung, esophageal, liver, ovarian, stomach and prostate cancer),* hypersensitivity (*Asthma, Atopy, Celiac disease, and dermatitis*), pregnancy complications (*Abortion, preeclampsia and premature birth*), vascular diseases (*Atherosclerosis, restenosis, and thromboembolism*), brain and mental diseases (*Depression, Migraine, Parkinson and Schizophrenia*) and metabolic complications (*Hypercholesterolemia, Obesity, Diabetes type 2* (DT2) and *metabolic syndrome*). **B** Differential Gene Expression of Genes represented by fold changes of all the CD genes, which show to be significantly different in at least one comparison. * denotes significance at q < 0.1 (holm *p*-value adjusted). Figures were mainly rendered in R software (https://cran.r-project.org/)

Regarding diseases, as expected, the most similar term to CD is IBD and UC, validating our strategy (Fig. 2). We observed a dense group of genes and diseases where CD is located close to other groups of autoimmune and inflammatory diseases, certain types of cancer, hypersensitivity disorders caused by allergies and intolerance, infections by virus and bacteria, pregnancy complications, and metabolic complications.

Gene sets from GO are divided into cellular components, biological processes, and molecular functions. Within biological processes terms, we observed significant enrichment in processes related to *response to bacteria*, *positive regulation of nitric oxide* (NO), *ERK*, and *NFκβ*, *apoptosis, cell proliferation, response to lipopolysaccharide*, *inflammatory* and *immune* response. For molecular function, *cytokine activity*, *interleukine-1 receptor binding, receptor activity, and protein homodimerization activity* were significantly enriched by the CD-risk genes analyzed. For cellular components, only *membrane, plasma membrane,* and *extracellular region* were significant*.* Overall the significantly enriched GO terms point to known CD terms such as the immune response, cytokine activity, and signaling receptors as the primary source of functional causes (in terms as *Immune Response, Infection, Interleukin-1 receptor binding, Cytokine and defense response, Cytokine activity, Autoimmune Disease*).

Additionally, among the enriched pathways identified were *JAK-STAT signaling pathway*, *cytokine receptor interaction*, *NOD-like receptor signaling pathway*, *NF-kappa*, *TNF signaling*, *Toll-like receptor (TLR) signaling, T cell receptor signaling*, and *Osteoclast differentiation*. These signaling pathways converge on the activation of NF-κB, a protein complex that controls the transcription of DNA cytokine production and cell survival [45]. In addition, the KEGG comorbidities identified are *infectious diseases* caused by *bacteria, protozoa and virus*, and *IBD,* which are reliable associations due to the relationship between microbes and CD [46]. The mapping is, therefore, an excellent guide to connect genes and important biological aspects of CD (Fig. 2).

Among the genes present in the enriched biological terms, we noted two distinct groups depending on the frequency of their presence in the gene sets and their number of abstracts found by PubTerm, designated as *common* and *sporadic* (Fig. 2)*.* Briefly, the 34 *common* genes are highly related to diseases and biological terms and well-studied. In comparison, the 92 *sporadic* genes are associated with particular diseases or biological terms and not as studied in CD as the *common* genes. Among the *common group,* the most shared genes across concepts are *NOD2, TNF, ICAM1 NFKBIA, NFKB1, TNFRSF1A, CD14, ACE TLR4,* and *TLR9,* which are involved in both TNF and of NF-κβ signaling pathways [47–49]*.* Also, *IFNG, IL1B, IL6, IL23R, IL10, IL4, IL12B, IL1RN, IL18,* and *IL4R,* which are all well-known cytokines or related genes, and contribute to the inflammatory response and cytokine interaction process [50–52]. The *sporadic* group comprised 92 genes that were much less frequent among enriched terms, where *ATG16L1, SLC22A4, IRGM, SLC22A5, TNFSF15, NOD1, PTPN2, PTPN22,* and *DLC5* being more frequently mentioned.

*DNAH12, ERAP2, FUT2, ORMDL3* and, *TRAIP* were more specifically enriched in the CD disease term and less common for the remaining terms.

**Fig. 3:**
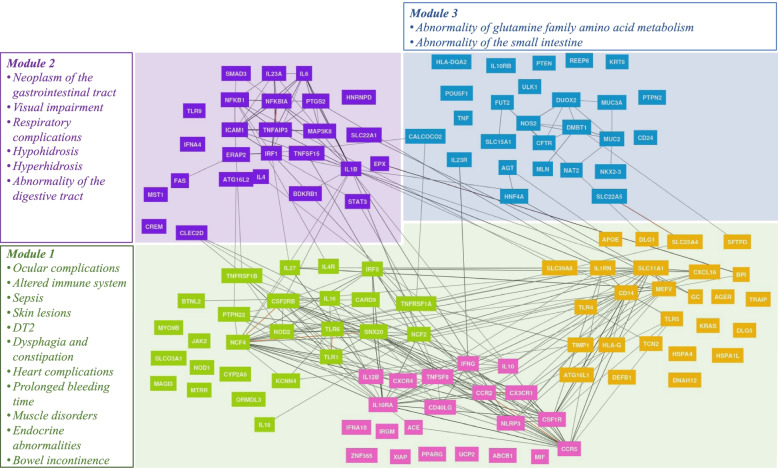
Network analysis for the three main groups identified. Group 1 (green): 73 genes, Group 2 (purple): 26 genes and Group 3 (blue): 27 genes. Figure adapted from Gene Network tool

Once we noted two clear sets of *common* and *sporadic* genes that were associated with specific terms, we considered whether the genes might also be grouped by other phenotypes that could explain CD symptoms. Thus, we used the Gene Network tool, which clusters genes with similar Human Phenotype Ontology. Five sub-networks were identified from the 126 genes categorized as *experimental evidence*. We then merged three highly interconnected sub-networks and subsequently analyzed the three resultant modules (73, 26, and 26 genes respectively for Module 1 in green, Module 2 in purple, and Module 3 in blue, as shown in Fig. 3). Next, for each module, the genes were also functionally analyzed to identify their potential phenotypic consequences. For this, we also used the Gene Network tool. Only exclusive terms for each module that were significant after Bonferroni correction were analyzed (see Methods).

For the first module (green), 60 phenotypes were retrieved; most of them related to severe symptoms such as *Ocular complications, Altered immune system, Sepsis, Bowel incontinence, Heart complications, Endocrine abnormalities, Dysphagia and constipation,* and *muscle problems*. For the second module (purple), 11 terms were identified mainly related to *Neoplasm of the gastrointestinal tract, Hyperhidrosis, Respiratory complications,* and *Visual impairment*. These symptoms are among common abnormalities detected in IBD patients [53–55]. Finally, for the third module (blue), only four consequences were identified, *abnormality of glutamine metabolism*, *abnormality of the small intestine,* and two remaining terms related to the *facial skeleton*. The information related to the module assigned to each gene is provided in Table S3.

### Gene expression analysis

A recent gene expression analysis of CD, UC, and controls in the colon and ileum showed that 1008 genes were differentially expressed [56]. We, therefore, explored whether the genes found genetically associated with CD in our systematic review were related to those differentially expressed (DE) between CD or UC relative to their normal ileum and colon gene expression. From the 126 genes, 67 genes were found to be DE for both UC and CD (Fig. 2B). The overlap between the 67 genes with those 1008 is highly significant (*p* = 1^− 290^, hypergeometric test), suggesting that our 126 genes are particularly enriched in DE genes. Except for *SLC39A8*, *FAS*, *IRF5*, *HSPA1L*, and *PTEN*, the vast majority were indeed more significantly associated with UC than with CD. Moreover, the majority of the genes were less expressed in CD relative to controls. We noted two solute carriers more expressed in CD than in normal colon or ileum; *SLC22A4* (ergothioneine, carnitine, tetraethylammonium) probably for detoxification, and *SLC22A5* (carnitine), whose variants are reported to affect the function of carnitine and organic cation transporters [57]. There are also two less expressed solute carriers, *SLC11A1,* with a role in the susceptibility of humans and animals to several infections [58] and *SLC39A8,* associated with gut microbiome composition [59].

### Drug-gene interaction analysis

The main therapeutic drugs for CD are azathioprine [60], infliximab, adalimumab, certolizumab pegol, ustekinumab, vedolizumab [61–63], prednisone, hydrocortisone, and hydrocortisone acetate [61]. To explore drug-gene interactions, an analysis was performed in DGIdb [41] using these drugs. There is a total of 78 genes that had a reported interaction with these CD therapeutic drugs, 10 of which were identified in our systematic review (Table 4). We reasoned that focusing on drugs that target the gene variants associated with IBD could be a strategy for CD drug repurposing [64]. Thus, to search for alternative drugs for CD, we used 13 other drugs commonly employed in the treatment of chronic autoimmune and inflammatory diseases [64, 65]. We identified 13 CD genes (IL1B, IL1RN, IL6, ABCB1, XIAP, IFNG, ICAM1, NLRP3, JAK2, PPARG, PTGS2, APOE, and SMAD3) that show some interaction and therefore could be further explored as possible treatments for CD (Table 4).

**Table 4:** Interactions between therapeutic drugs and the 126 genes

***Disease***	***Drug***	***Gene interaction***
*Crohn’s disease*	Infliximab	*TNF*, *TLR4*
	Prednisone	*APOE*, *IFNG*, *ABCB1*
	Hydrocortisone	*NOS2*, *ABCB1*, *IL1B*, *AGT*
	Adalimumab	*TNF*
	Ustekinumab	*IL12B*, *IL23A*
	Certolizumab pegol	*TNF*
	Azathioprine	–
	Hydrocortisone acetate	–
	Vedolizumab	–
*Chronic autoimmune and inflammatory diseases*	Canakinumab	IL1B
	Rilonacept	IL1B, *IL1RN*
	Metronidazole	*IL6*
	Rituximab	*ABCB1, XIAP*
	Methylprednisolone	*ABCB1, IFNG*
	Methotrexate	*IL1RN*
	Natalizumab	*ICAM1*
	Anakinra	*NLRP3*
	Olsalazine	*IFNG*
	Tofacitinib	*JAK2*
	Sulfasalazine	*PPARG, PTGS2*
	Triamcinolone	*APOE*
	Dexamethasone	*SMAD3*

### Comparison of genetic panels

To compare the generated list of genes with genetic panels already in use, we benchmarked within those panels in the GTR [42]. We found 21 panels, of which 19 were specific to Crohn’s disease containing only 2 genes, *NOD2* and *IL6*. The two remaining tests were not specific for Crohn’s, IBD, and related diseases. These tests considered 70 genes, of which 22 were identified as functional variants for CD in our curation (including *NOD2* and *IL6)*. Thus, from the 256 genes we found (Tables 3 and S3), 225 genes were not included in any panels for CD or IBD-related disorders.

We also verified the identifications at the Open Targets (OT) platform, whose pipeline includes a fine mapping of variants [31]. Filtering 3093 genes for CD for genetic association score > 0.8, 178 genes were identified. Of these, 3 genes (SNN, SH2B3, and SKAP2) were not identified in the set of 1092 curated genes. From the 126 genes identified here having experimental evidence, 39 showed a low genetic association score for CD (0.5 to 0.79), 8 genes showed a good score (0.8 to 1.0) for IBD, and 52 genes do not have genetic data information in OT for CD nor IBD. Some of the 52 genes show variations that have not been reported in GWAS, explaining their absence in OT. Examples include *NOD1*, *ABCB1*, *IL1RN*, *MEFV*, and *IL18* having variants that could not be easily identified in GWAS because there are triallelic changes [66], deletions [67], and VNTR [68]. This comparison shows that even GWAS information can leave aside some information of other variants detected through other technologies or methodologies.

## Discussion

Through our methodology, we have identified 256 genes associated with some aspects of CD. Of them, 126 genes were associated with *experimental evidence of variants in CD*, 71 genes found in *GWAS with a sequence variant within the gene*, 41 genes for *complications*, and 18 genes for *treatment response in CD.*

There is an explosion of genetic data provided by the high throughput technologies such as genome-wide SNP arrays and next-generation sequencing. This growing list of associated genes has the potential to improve diagnosis and treatment, but progress has been slow. There is a need for better strategies for prioritization and curation. In this study, we found that from 126 genes with variants associated with CD, a total of 110 genes have not been included in any genetic panel for CD and related diseases. That is probably reflected by the lack of individual predictive value of most individual common SNPs. The small number of variants annotated in ClinVar [32] seems to be caused by some variants not found by GWAS. Also, the increasing tendency of acquiring genetic data suggests that more efforts and more accurate annotations, such as those provided here, are highly needed and valuable.

### In-silico functional analysis

The functional bioinformatics analysis performed confirmed the relationship between CD and highlighted modules of genes in our systematic review. We identified autoimmune diseases that could have affected pathways similar to those of CD such as *Type 1 Diabetes*, *Multiple sclerosis*, *Lupus*, A*rthritis Rheumatoid*, and *Psoriasis.* These relationships among CD and other autoimmune diseases are already known and have been previously studied [69, 70]. There are also relationships with hypersensitivity diseases such as asthma and celiac disease and metabolic complications such as T2D and hypercholesterolemia. Indeed, there are some Previous studies have shown an association of CD and IBD with asthma [71], type 1 diabetes [69], and T2D [72]. Those diseases identified in our analysis are likely to share a genetic background with CD due to their inflammation process and their condition as autoimmune diseases, as suggested by previous studies [69, 71, 72]. Our results highlight the genes which could be shared among conditions and allow focusing on future research efforts among these genes.

Additionally, we found functions related to immune response, cytokine activity, and receptors. It is clear that CD pathogenesis is caused by an immune imbalance [73], which was also reflected in our de novo analysis. Some hypotheses have attempted to explain its mechanisms, including delayed hypersensitivity, activation induced by food, and others [73]. These mechanisms converge into the immune response in an environment where self-tolerance has been lost and where cytokines have an active role in maintaining this pro-inflammatory state [73, 74]. Additionally, other terms, such as apoptosis and response to Lipopolysaccharides (LPS), may provide interesting insights. LPS response is related to a monocyte/macrophage stimulation by enteric bacteria constituents [75], and resistance to apoptosis in patients with CD has also been reported [76].

We spotted signaling pathways specific for some important genes in CD converging on the activation of NF-κB, which is a protein complex that controls the transcription of DNA cytokine production and cell survival [45]. This is comparable with previous reports of abnormal activation of NF-κB, causing chronic inflammation in the bowel [45]. Similarly, pathways related to infections caused by protozoa, virus, and bacteria were identified consistent with the known relationship between microbes and CD [46]. Pathogen infections are one of the environmental factors which are likely to be a key component for CD; however, their roles or mechanisms of action remain speculative [77]. Additionally, the microbiota plays an important role [56]. Our results show that most of the genes related to pathogen infections are among the *common* genes and close to the pathways of *NOD*, *TLR*, and *NFκβ,* which could aid in the future understanding of the mechanisms of action specifically in CD. We also observed the pathway for *Osteoclast differentiation*, which has been recently studied, linking the function of *IL-17*, and *TNFa* modulating bone resorption [78].

In our functional analysis, we spotted the following genes, *NOD2, IL23R, IL6, IRGM, ATG16L1,* and *IL10,* whose CD-predominant risk associations are known [48, 79–82]*.* Among them, *NOD2* has the highest contribution to CD risk alone, with 5% of penetrance and ~ 20 fold risk [82]. Other genes that are not currently present in any diagnostic test for CD or a related condition but which showed general importance for CD, related diseases, and biological process, molecular function, and pathways are *TNF, ICAM1, NFKBIA, NFKB1, TNFRSF1A, CD14, ACE, TLR4, TLR9, IFNG, IL1B, IL4, IL12B, IL1RN, IL18,* and *IL4R,* which are involved in TNF and NF-κB signaling pathways, in inflammatory response, and cytokine interaction processes [47–52]. These genes and others from our list could be used to design a more robust prediction panel for CD risk.

Our analysis highlighted poorly studied genes (10 or fewer abstracts). From these, FUT2, *DNAH12, TRAIP,* and *ERAP,* were identified in the functional analysis to be specific for CD (Fig. 2). Among the processes reported for these genes are ABH antigens expression [83], motile cilia function [84], regulation of innate immune signaling [85], and immune activation and inflammation [86].

Among these poorly studied genes, only *FUT2* is currently present in a diagnostic panel related to IBD diseases. This fact remarks the importance of considering and further studying the biological implication of the less studied set of genes to increase our knowledge of this complex disease.

### Network analysis

We identified three network modules of genes associated with specific symptoms. The first module comprising 73 genes was related to severe symptoms [87], such as *Altered immune system, Sepsis, Bleeding, Muscle disorders*, and *Heart complications* well-known in CD. The second module involved 26 genes related to *hyperhidrosis*, *respiratory complications*, and *neoplasm of the gastrointestinal tract*. These symptoms are among the common abnormalities detected in IBD patients [53–55]. The third module, including 26 genes, was associated with *abnormality of glutamine metabolism* and *abnormality of the small intestine*. Glutamine is an important supplementation in IBD patients [88], and its effects in IBD have been studied in animal models [89] and patients [90, 91]. Thus, this module seems to map genes related to less severe consequences for CD. Thus, gene-symptom mapping may provide important insights into CD.

### Gene expression analysis

Differential expression analysis of the 126 genes identified in our systematic review revealed that a significant number of genes show dysregulated levels of expression in colon and ileum biopsies of both CD and UC when compared with not-IBD patients further supporting our gene prioritization approach. The greatest changes in expression are observed in the colon, with differential expression in pro-inflammatory genes (*NOD2, IL1B*, and *TNF*). Other observations are that changes are different among UC and CD and that a large proportion of the genes do not show evident gene expression. Thus, to further understand whether the functional implications of these changes in expression are causal for CD pathogenesis or whether CD patients carrying other specific variants show different gene expression profiles, further functional experiments are needed.

### Drug-gene interactions

Among the 126 genes analyzed, 10 have a reported interaction with known CD therapeutic drugs, and 13 have a reported interaction with other autoimmune and inflammatory diseases. The treatment for CD is complex, and it is focused on controlling the symptoms and the remission of the disease [92]. Focusing on drugs that target the gene variants associated with IBD can be a strategy for CD drug development [64]. This highlights the necessity of considering more genes to study other possible interactions for CD beyond what is currently known and shows the importance of gene curation strategies, like the one proposed here. Further research on the genes highlighted here, and their mechanisms of interaction with CD diseases could improve the knowledge of the disease development and expand treatments.

Traditional drug development is costly and can take 10–15 years to develop an efficient drug [64]. Personalized medicine exhibits the clinical application of drug-gene interaction, where drugs are guided based on the individual’s genetics and disease progress. Targeting CD’s genetic risk regions that had been experimentally validated can improve the identification of possible drug candidates. This can be reflected in target-directed therapies, which is one of the main objectives of personalized medicine. The analysis of drug-gene interactions in a complex disease, such as MDD (major depressive disorder), allowed a better, prioritization of drug-genes sets and the identification of drugs indicating an effect on a disease, reflecting potential repurposing opportunities [93]. Nevertheless, validation studies are still required to ensure the drug-gene interaction and avoid side effects.

Our results support the consideration of several genes when studying CD. More importantly, the functional analysis provides a mapping between genes and key aspects of Crohn’s disease. The integration of other genes may also be important. For example, genes close by a non-coding GWAS SNP, i.e., intergenic variants, or those involved in related diseases, could play a role in CD etiology, but further validation or fine gene mapping is needed.

## Supplementary Information

**Additional file 1.** PRISMA checklist.

**Additional file 2.** List of Pubmed IDs for genes curated trough PubTerm.

**Additional file 3.** List of Pubmed IDs for genes curated from GWAS Catalog.

**Additional file 4: Table S1.** Annotations for more than 1000 genes for their association to CD.

**Additional file 5: Table S2.** Annotations for 133 genes from GWAS Catalog associated to CD.

**Additional file 6: Table S3.** Details of the 126 genes categorized as experimental evidence for CD.

**Additional file 7: Table S4.** Mutations of associated to genes not annotated in other databases.

**Additional file 8: Table S5.** Annotation for genes categorized as other genetic associations.

## Data Availability

The prioritized gene catalog can be explored at http://victortrevino.bioinformatics.mx/CrohnDisease.

## Abbreviations

CD: Crohn’s disease
IBD: Inflammatory Bowel Disease
UC: Ulcerative Colitis
GWAS: Genome-Wide Association Studies
SNP: Single Nucleotide Polymorphism
NGS: Next-Generation Sequencing
PRS: Polygenic Risk Score
GAD: Genetic Association Database
GO: Gene Ontology
HPO: Human Phenotype Ontology
DGIdb: Drug-Gene Interaction Database
GTR: Genetic Test Registry
